# *In Silico* Study of Rotavirus VP7 Surface Accessible Conserved Regions for Antiviral Drug/Vaccine Design

**DOI:** 10.1371/journal.pone.0040749

**Published:** 2012-07-26

**Authors:** Ambarnil Ghosh, Shiladitya Chattopadhyay, Mamta Chawla-Sarkar, Papiya Nandy, Ashesh Nandy

**Affiliations:** 1 Physics Department, Jadavpur University, Kolkata, West Bengal, India; 2 Division of Virology, National Institute of Cholera and Enteric Diseases, Kolkata, West Bengal, India; 3 Centre for Interdisciplinary Research and Education, Kolkata, West Bengal, India; Hallym University, Republic of Korea

## Abstract

**Background:**

Rotaviral diarrhoea kills about half a million children annually in developing countries and accounts for one third of diarrhea related hospitalizations. Drugs and vaccines against the rotavirus are handicapped, as in all viral diseases, by the rapid mutational changes that take place in the DNA and protein sequences rendering most of these ineffective. As of now only two vaccines are licensed and approved by the WHO (World Health Organization), but display reduced efficiencies in the underdeveloped countries where the disease is more prevalent. We approached this issue by trying to identify regions of surface exposed conserved segments on the surface glycoproteins of the virion, which may then be targeted by specific peptide vaccines. We had developed a bioinformatics protocol for these kinds of problems with reference to the influenza neuraminidase protein, which we have refined and expanded to analyze the rotavirus issue.

**Results:**

Our analysis of 433 VP7 (Viral Protein 7 from rotavirus) surface protein sequences across 17 subtypes encompassing mammalian hosts using a 20D Graphical Representation and Numerical Characterization method, identified four possible highly conserved peptide segments. Solvent accessibility prediction servers were used to identify that these are predominantly surface situated. These regions analyzed through selected epitope prediction servers for their epitopic properties towards possible T-cell and B-cell activation showed good results as epitopic candidates (only dry lab confirmation).

**Conclusions:**

The main reasons for the development of alternative vaccine strategies for the rotavirus are the failure of current vaccines and high production costs that inhibit their application in developing countries. We expect that it would be possible to use the protein surface exposed regions identified in our study as targets for peptide vaccines and drug designs for stable immunity against divergent strains of the rotavirus. Though this study is fully dependent on computational prediction algorithms, it provides a platform for wet lab experiments.

## Background

Rotavirus, a dsRNA virus of Reoviridae family causes severe diarrhea in children below five years of age. Every year an estimated 527,000 children die from rotavirus diarrhea, with more than 85% of these deaths occurring in developing countries of Africa and Asia [1]. Rotaviruses primarily infect the enterocytes of the small intestine, but it is also found to be associated with Central Nervous System (CNS) complications. From 1975 through 2000 there were 12 case reports where rotavirus was noted along with CNS complications [2].

Rotaviruses are divided into seven groups based on their antigenic specificity and designated A, B, C, D, E, F and G. Though Group A is the most commonly found pathogenic rotavirus, group B and C rotaviruses can also be found in some human infections. The rotavirus genome consists of eleven segments of dsRNA which encode for twelve proteins of which six are nonstructural (NSP1-NSP6) (Non Structural Protein) i.e. they only appear after infecting the host cells, and other six are structural proteins (VP1-VP4, VP6 and VP7) [3]. Most of the outer capsid is made of the 37 kDa VP7 glycoprotein, the other surface protein being the nonglycosylated protein, VP4. VP4 is present on the virion in the form of spikes while the VP7 covering almost the entire virion is 6.5 times more abundant [4].

Two different kinds of serotype (genotype) specificity, namely G and P were designated for Group A rotaviruses (GARV). The G and P serotypes are based on antigenic specificity (now by sequence homology), representing Glycosylated VP7 (G type) and Protease sensitive protein VP4 (P type) [4]. These two proteins have been the target of vaccine preparation for many years because of their surface exposed structure and considerably large size making them good targets for neutralizing antibodies that give serotype specific as well as in some cases cross-reactive protection. There are 23 G types and 31 P types identified in GARV at present [5]. Since GARVs not only have multiple hosts (humans and many farm animal species) but also have segmented genome, the frequency of reassortment and interspecies transmission to form novel serotypes is high [6]. The mutation rate and genetic variation of rotavirus genes including VP7 are considerably high, therefore continuous modifications in genotyping primers are required to prevent genotyping failure [7]. Due to both genetic and antigenic heterogeneity, there has been an on-going debate whether any monovalent vaccine will give protection against all G and P types as well as against unusual strains which originate due to interspecies transmission as these are more likely to cause outbreaks [8].

**Table 1:** Assignment of axis to individual amino acids.

Axis No.	Amino acid	3-letter code	Single letter code	Axis No.	Amino acid	3-letter code	Single letter code
1	Alanine	Ala	A	11	methionine	met	M
2	Cysteine	Cys	C	12	Asparagine	asn	N
3	aspartic acid	Asp	D	13	Proline	pro	P
4	glutamic acid	Glu	E	14	Glutamine	glu	Q
5	Phenylalanine	Phe	F	15	Arginine	arg	R
6	Glycine	Gly	G	16	Serine	ser	S
7	Histidine	His	H	17	Tyrosine	tyr	T
8	Isoleucine	Ile	I	18	Valine	val	V
9	lysine	Lys	K	19	Tryptophan	trp	W
10	Leucine	Leu	L	20	Tyrosine	tyr	Y

Although no effective drugs have been developed to date, there have been several vaccine attempts made along the years, most of which were live attenuated and therefore always carried a threat of reversion to a virulent form. Many vaccine attempts failed at the preclinical and Phase-I clinical trials due to one or more reasons. The first rotavirus vaccine to be licensed for use in the USA was Rotashield, a Rhesus Rotavirus tetravalent (RRV-TV) licensed in the year 1998 for oral administration. After 9 months of the start of the vaccination program the vaccine was withdrawn from the market because of observed complications of intussusception, i.e. the invagination of the intestine into a distal segment. Within the initial stages of study it was estimated that 1 in 2500 to 1 in 3350 cases of intussusception risk was associated with the vaccine. In the USA alone, 98 confirmed cases of intussusceptions were reported within August 1999 which finally led to suspension and eventually withdrawal of the vaccine from the market [9]. A combination of all these factors has resulted in delay in development of effective vaccine in spite of continuing efforts since 1970s.

**Figure 1:**
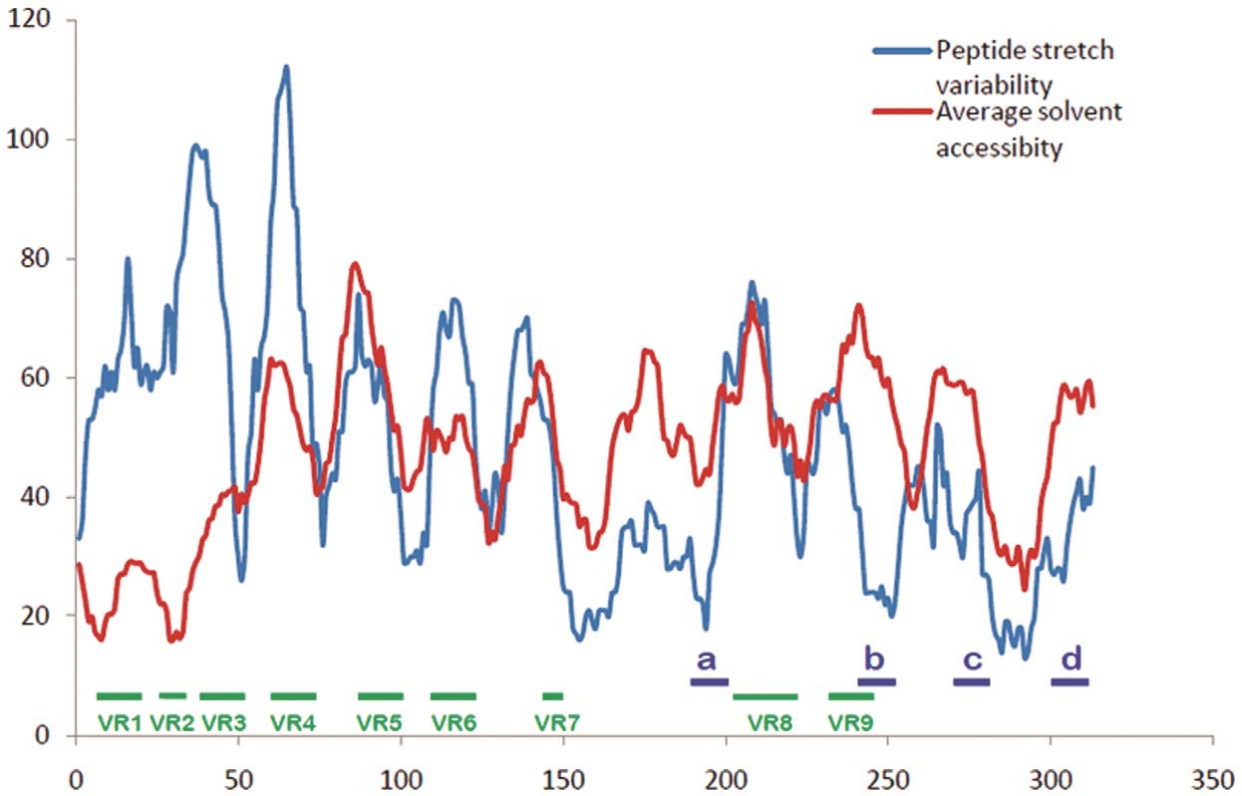
Comparison of solvent accessibility and stretch variability in rotaviral VP7 sequence database. Comparative diagram between solvent accessibility and stretch variability shows the regions of lowest variability and greatest environmentally accessed. All nine variable regions documented from previous research are found clearly distinguishable through our graph. Those regions are marked in green line. Peptide regions (peptide-a, b, c & d) indentified by our method are marked by blue lines.

**Figure 2:**
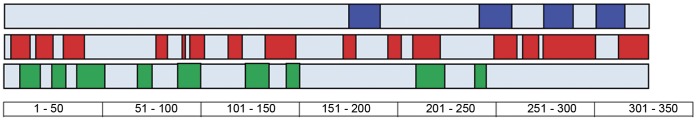
Comparative representation of conserved, variable and antigenic regions from rotaviral VP7 protein database. Comparative schematic diagram for conserved surface accessible region (blue), previously recorded antigenic regions from IEDB server (red) and variable regions of rotavirus (green). Here length of protein VP7 is 326 amino acids.

Another concern associated with oral rotavirus vaccines is the possibility that vaccines which give protection against children of developed countries of America and Europe might not work properly in the children of the developing world. The first developed Rotavirus vaccine RIT 4237 (1984) gave effective protection in Finnish children but failed to give the same in children of Rwanda and Gambia. This is in consonance with the observations that live oral vaccines of polio and cholera were also found to be much less effective in developing countries such as India than in countries of North America and Europe [10]. Similarly currently licensed rotavirus vaccines exhibited high efficacy (∼85%) against severe rotavirus diarrhea in industrialized nations, but shows only ∼40–60% efficacy in developing nations [11].

**Figure 3:**
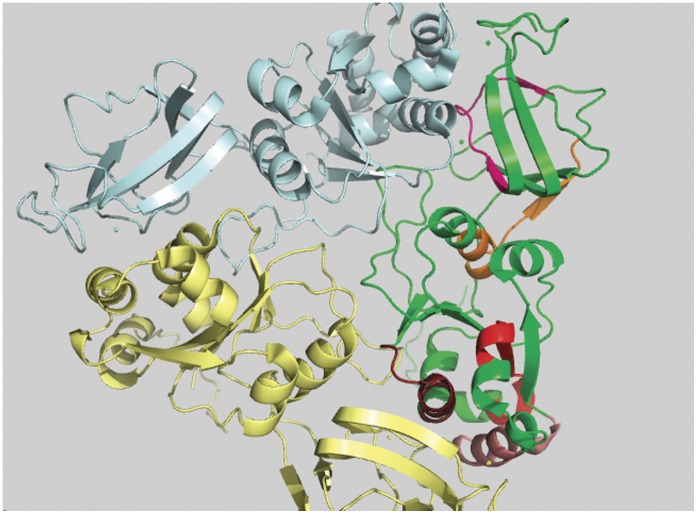
Conserved and surface inaccessible regions of rotaviral VP7 protein. Five conserved and surface inaccessible regions are shown in five different colors and they are represented in green colored monomer of trimeric VP7 protein.

**Figure 4:**
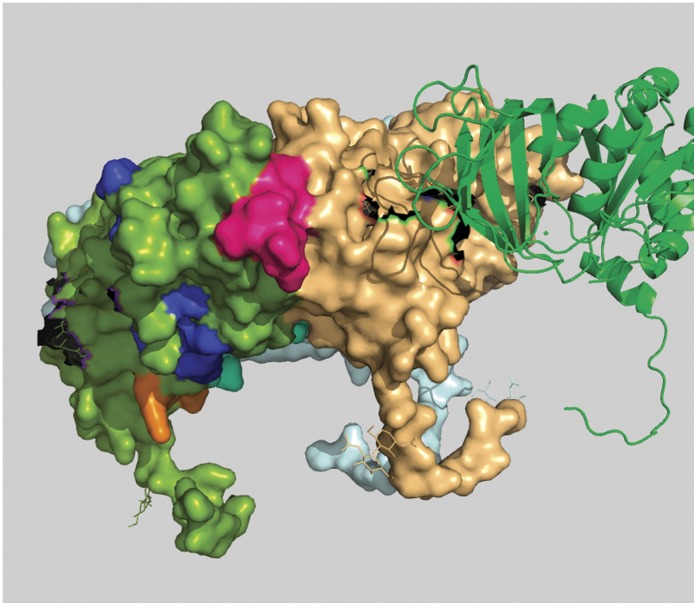
Position of peptide-a in a 3D space filling model of rotaviral trimeric VP7 protein. Peptide-a (194aa to 207aa) is shown in bright pink color and it is positioned in the green colored monomer.

Currently two vaccines have been licensed and approved by the US Food and Drug Administration (FDA), RotaTeq and Rotarix, and have been included in EPI (Expanded Programme on Immunization) vaccine schedule in many countries. These two vaccines have done well in clinical trials in reducing the episode of childhood rotavirus diarrhea mainly in developed countries like Finland where RotaTeq exhibit 84% reduction in emergency hospitalization due to rotavirus diarrhea. Rotavirus vaccine efficacy is very low in underdeveloped countries like India, Bangladesh, Kenya, Mali and Ghana [10]. The expectation from live, oral rotavirus vaccines was that they should protect against all cases of rotavirus diseases of any severity, just like one natural infection which provides 87.1% protection against severe disease and almost 100% after two infections [12]. The goal could not however be achieved in third world nations with high death but low efficacy of live oral vaccines.

**Figure 5:**
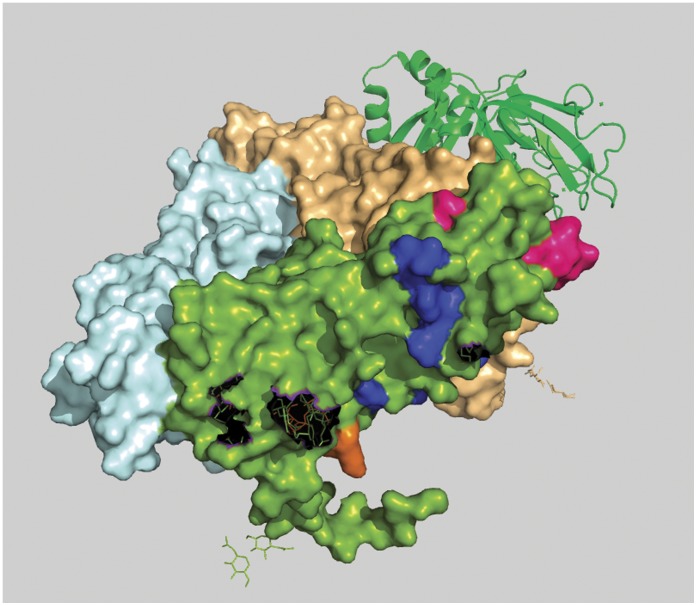
Position of Peptide-b is shown from outer surface of the virion in 3D-space filling model of rotaviral trimeric VP7. Peptide-b (aa 242 to aa 255) is shown in blue color and clearly highlights its discontinuous characteristic.

**Figure 6:**
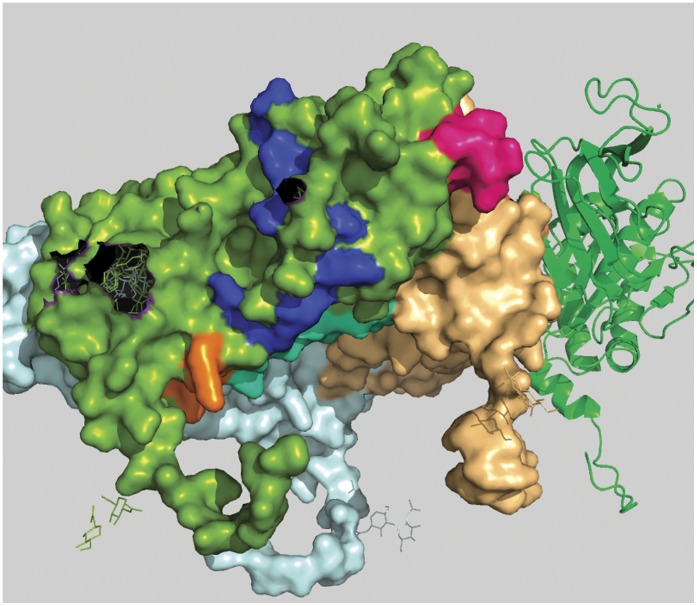
Position of Peptide-b is shown from inner surface of the virion in 3D-space filling model of rotaviral trimeric VP7. Lower part of Peptide-b (aa 242 to aa 255) is shown in blue color. It’s the same blue peptide as in Figure 5.

In-addition the Rotarix vaccine was recently found to be contaminated with porcine circovirus (PCV1) viral DNA and was temporarily suspended by US FDA in March 2010. Later, PCV1 and PCV2 DNA were also found in the other licensed vaccine RotaTeq. However it was proven that porcine circovirus did not impose any threat to human and thus both vaccines have been declared safe for use [13]. But this still questions the safety of tissue culture generated vaccines which may have higher chances of contamination during the manufacturing process.

**Figure 7:**
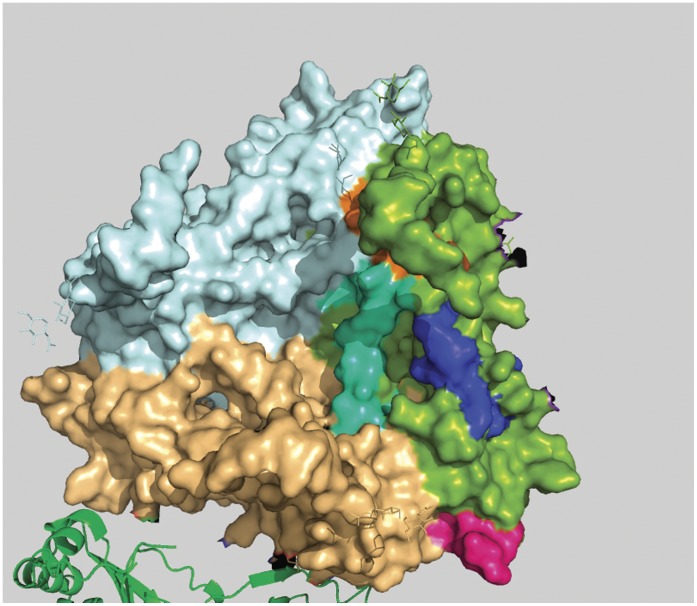
Position of peptide-c in a 3D space filling model of rotaviral trimeric VP7. Peptide-c (273 aa to 286 aa) is shown in cyan color and it is positioned in the green colored monomer.

Based on this observation as well as unresolved safety concerns associated with live virus vaccines, alternate strategies are being used to develop next generation candidates. These range from fully inactivated rotavirus, DNA vaccines, recombinant E. coli expressed rotavirus proteins etc. Inactivated rotaviruses administered either intra-nasally, peritonally or intra-rectally have shown good protection following rotavirus challenge in mice [14]. VP6 protein expressed in E. coli also was found to protect mice against fecal rotavirus shedding [15]. The level of protection has been 90% and was heterotypic. Unlike intestinal IgA which is considered the primary effector after oral immunization, mucosal (intestinal) immunization with VP6 activated CD4+ T cells, suggesting a different mechanism of action between two classes of vaccines.

**Table 2:** MHC-II binding results for the identified four peptide segments.

PeptideSegmentNumber	Allele	Position	Sequence	arb_core	arb_score	arb_percentile	smm_core	smm_score	smm_percentile	Consensuspercentile
**A**	HLA DPA1*01-DPB1*0401	1∶193–207	VKVCPLNTQALGIGC	VKVCPLNTQ	4620.916471	39.42	LNTQALGIG	14895	80.96	60.19
	HLA DPA1*01-DPB1*0401	1∶194–208	KVCPLNTQALGIGCQ	PLNTQALGI	7408.864636	57.03	LNTQALGIG	16291	83.41	70.22
**B**	HLA DPA1*01-DPB1*0401	1∶241–255	TTTCTIRNCKKLGPR	RNCKKLGPR	55554.40475	99.04	IRNCKKLGP	35395	96.59	97.815
	HLA DPA1*01-DPB1*0401	1∶242–256	TTCTIRNCKKLGPRE	NCKKLGPRE	19175.64294	87.39	IRNCKKLGP	35871	96.7	92.045
**C**	HLA DPA1*01-DPB1*0401	1∶272–286	TADPTTNPQIERMMR	TADPTTNPQ	5420.43634	45.2	TTNPQIERM	25803	92.94	69.07
	HLA DPA1*01-DPB1*0401	1∶273–287	ADPTTNPQIERMMRV	ADPTTNPQI	5654.176164	46.76	TTNPQIERM	18235	86.23	66.495
**D**	HLA DPA1*01-DPB1*0401	1∶300–314	VDYINQIVQVMSKRS	VDYINQIVQ	4015.810591	34.58	INQIVQVMS	6833	53.94	44.26
	HLA DPA1*01-DPB1*0401	1∶301–315	DYINQIVQVMSKRSR	DYINQIVQV	7495.055799	57.47	INQIVQVMS	8788	63.44	60.455

Results of the peptide-a, b, c and d submitted to the IEDB T-cell MHC-II binding prediction server are tabulated here. For each peptide the server generates two overlapping peptides; the peptides for the four groups are shown in different colors.

Immunogenicity studies of rotavirus action have shown that both the surface proteins, VP7 and VP4, are immunogenic under experimental conditions and during natural infections [16]. Neutralizing antibodies interact with the outer surface protein of rotavirus, VP4 and VP7, and the most efficient rotavirus-neutralizing antibodies are reported to be all directed against the VP7 [17]. Ruggeri and Greenberg [16] found through monoclonal antibody (MAb) studies that VP7 specific MAbs (Monoclonal antibodies) are more efficient in neutralizing viral infection than VP4-MAbs due mainly to the larger concentration of the VP7 proteins on the virion surface: total VP7 protein molecules (780) present are 6.5 times more than VP4 protein molecules (120) in every virus particle [4]. Their studies indicated that antibodies directed at the VP8* fragment, a trypsin digestion product of the VP4, appear to inhibit binding of the virus to the cells, whereas antibodies directed at the VP7 was determined by Ludert et al [17] to neutralize by inhibiting virus decapsidation and therefore not all molecules of VP7 need to be combined with antibodies to achieve neutralization. Experimental evidence of Ruggeri and Greenberg also suggests that VP7-MAbs can mediate dramatic decrease in viral infectivity, which is markedly less in case of the VP4-MAbs. Further, rotavirus VP7 protein has been shown to trigger polyclonal B-cell activation without the presence of viral RNA, or VP4 protein [18]. In this paper an attempt has been made to primarily identify conserved peptide sequences in the VP7 surface protein. Such a study is indicated by the importance of the VP7 in rotaviral immunogenicity, and also by the analysis of Aoki et al [19] that in all 11 human G serotypes they studied; most of the variability was on the outward-facing surface of the VP7 trimer, whereas efficient antibody binding would require surface exposed residues. We have also done similar analyses for the VP4 and VP6 protein sequences and the results are presented separately.

**Table 3:** Tabulated from of results derived from ABCpred server.

Rank	Sequence	Start position	Score
**1**	**TTTCTIRNCKKLGP**	**241**	**0.92**
**2**	**LDITADPTTNPQIE**	**269**	**0.89**
**2**	**KVCPLNTQALGIGC**	**194**	**0.89**
3	SDGEWKDSLSQMFL	94	0.87
3	SFETVAENEKLAIV	214	0.87
4	LMKYDQNLELDMSE	141	0.85
5	ISVALFALTKAQNY	40	0.84
5	TTNVDSFETVAENE	209	0.84
6	KLAIVDVVDGINHK	223	0.83
7	LCLYYPTEASTQIS	81	0.82
8	IFLTSTLCLYYPTE	75	0.81
9	IPITGSMDTVYSNS	57	0.8
**9**	**KINLTTTTCTIRNC**	**236**	**0.8**
10	LGIGCQTTNVDSFE	203	0.78
11	SGESNKWISMGSSC	178	0.77
12	SELADLILNEWLCN	153	0.76
13	ITLYYYQQSGESNK	170	0.75
14	ALTKAQNYGLNIPI	46	0.73
15	WLCNPMDITLYYYQ	163	0.72
**16**	**RNCKKLGPRENVAI**	**247**	**0.7**
16	FLISIILLNYILKS	12	0.7
17	SNSTQEGIFLTSTL	68	0.69
17	QNLELDMSELADLI	146	0.69
18	YRFLLISVALFALT	35	0.67
18	LYCDYNLVLMKYDQ	133	0.67
18	QMFLTKGWPTGSVY	104	0.67
19	ILKSVTRIMDYIIY	22	0.66
20	YSNIVDFSVDPQLY	121	0.62

The bold rows show that the conserved surface exposed regions identified in our method are also covered in five of the ABCpred server predicted regions.

It has been noted that universal vaccine and cross protective vaccine can bring broad spectrum disease protection against highly mutating viruses like influenza, HIV, rotavirus, etc. [20], [21], [22], [23]. The idea for such type of vaccine design is based on the evolutionarily conserved portion of the viral protein which can act as an epitope or candidate of antibody recognition [22]. If it is possible to design antibody or small molecule that recognize those conserved regions, the protein can be inhibited and the virus can be controlled.

To determine such regions in the rotavirus surface proteins, we use a graphical representation method for analysis of protein sequences [24]. Graphical representations introduced by Nandy, Randic, Leong and others in biology to represent of DNA/Protein sequences have been reviewed in detail by Randic et al. [25] and extended to many classes of systems by Gonzalez-Diaz et al [26]. In general they perform a pseudo-folding process of the sequences into 1D, 2D, 3D, 4D or even higher dimensions (top in 20D) spaces embedding sequences on rectangular, zigzag, lattice, star, or spiral-like patterns among others [27], [28], [29], [30], [31], [32], [33], [34]. One of the great advantages of the method, apart from elegant and compact visual representations, is the possibility of calculating numerical parameters of these graphs. These parameters can be used to numerically compare different systems or even can be applied to the classic ideas behind QSAR analysis of small molecules to very complex biosystems [35], [36], [37], [38], [39]. We had used graphical representation and numerical characterization (GRANCH) methods to identify surface situated conserved regions in the neuraminidase protein of H5N1 influenza virus [40], [41]. In this paper we use a modified and updated GRANCH technique [42] to search for similar regions in the rotavirus proteins and further analyze for epitopic possibilities.

## Materials and Methods

### Database

We have considered a database consisting of 438 complete sequences (accessed 1^st^ November 2010) of rotaviral surface glycoprotein VP7 of common serotypes G1 to G15, G20 and G21 from mammalian hosts. We specifically excluded the sampling from the serotype G19, G22 and G23 because they impact avian sources only. All these sequences are retrieved from the NCBI viral genomic database. Out of the 438 complete VP7 sequences, we have restricted our analyses to 433 sequences that have 326 amino acids each making up the complete sequence. 5 sequences originating from avian hosts with amino acid lengths exceeding this common length were discarded.

We have also accessed 100 sequences each of VP4 and VP6 proteins from the NCBI database for the complementary analyses restricting the list to equal length sequences (775 residues for the VP4 and 397 for the VP6) (File S1).

### Method of Analysis

We had earlier described a technique to analyze a protein database to determine surface exposed conserved peptide segments by using the gene and amino acid sequences, the solvent accessibility profile and the protein 3D structure data. As mentioned earlier, for the rotavirus we use the VP4, VP6 and VP7 protein sequences for analysis. The individual components of the analysis are described briefly below.

### 20D Protein Sequence Representation

The method is essentially based on a protein graphical representation and numerical characterization technique developed by our group following the 2D DNA sequence representation and characterization method [24]. To characterize protein sequences graphically, we model a protein sequence in the abstract using a 20-dimensional Cartesian coordinate system [24]. We associate each amino acid with one axis of the 20D Cartesian coordinate system; the choice of association is equivalent for all residues, but once assigned will be fixed for the duration of the computation. For easy computation and comparison we have calculated weighted averages and resultant vectors that are unique to the respective sequences as in the case of the nucleotide sequence representations. Briefly, we assign amino acids to the 20 axes [24] as in **Table 1**.

Thus, a sequence like MVHLTPEEKS will have an end-point coordinate as (0,0,0,2,0,0,1,0,1,1,1,0,1,0,0,1,1,1,0,0). We can then define a weighted mean c.m. µ*_i_* for each axis *i* and a graph radius *p_R._*


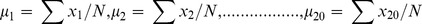



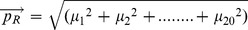


which constitute the protein graph descriptors. The *p_R_* are quite sensitive to amino acid changes in sequence comparisons [24], [43]. Here this technique is used to compute the protein descriptors to determine similarity/dissimilarity between different protein sequences including short protein stretches.

#### Adjustments to computation of *p_R_*

The symmetry of the computation method for the residues could give rise to an apprehension that degeneracies could result in some cases where different sequences could lead to the same values of the *p_R_’*s. This can be most easily seen if one considers two homologous sequences where a residue that occurs only once at a position in the sequences is replaced by another residue occurring only once in the second sequence, but again at the same position. There could also be the possibility of different combinations of residues leading to degeneracy. The chance of such happenings in full protein sequences would be remote, but could happen when comparing small peptides. While this has not occurred in the exercise at hand for this paper, for completeness we need to find ways and means to take care of this problem.

The point to note is that the absolute value of the *p_R_* has not been associated with any property of the protein sequences; the *p_R_*s are used only in a relative sense to compare two or more sequences or peptides. Thus suitable modifications to determining the *p_R_*, applied equally to all sequences under investigation would serve the purpose of inter-sequence comparison. We use this principle in two ways: The first method is to start from the end of the unit cube, i.e. the co-ordinate position (1,1,1,…,1) to computing the *p_R_* [42]. This adds the necessary padding to remove the second class of problems mentioned above. The second method is to introduce an asymmetry in the computation by adding a small but different number to each axis; we have used a sequence of .001, .002, .003, …, .02 to the 20 co-ordinates. One could also combine both modifications to ensure best adjustment for the computation of the *p_R_*.

### Graphical Sliding Window Method (GSWM)

The calculation of GSWM is done on a 20 dimensional Cartesian coordinate system [43]. In this process the entire sequence is subjected to a shifting window scan where the protein descriptor, *p_R_*, is measured for the peptide stretch covered by the window, and the next *p_R_* value is generated by shifting the window by one position and so on until the complete length of the sequence is covered. This is done for every rotavirus sequence in the database and the number of different values of the *p_R_* that results at each position is determined and plotted leading to a variability profile from the overlapping windows [40].

It is instructive to note here that in our method of analyzing all amino acid sequences we do not require any multiple alignment, and computation of *p_R_* values for window sizes of, e.g., 8 or 10 or 14 amino acids, can always be done contiguously.

To determine an appropriate window size, while we have used 8 to 12 amino acid window size for our previous analysis of the neuraminidase protein, in view of the reported high variability of the rotavirus, it was considered more appropriate to choose window sizes of 8 to 14 for the current analysis. Our choice of 14 amino acid window size in this study is also based on previous reports that a 14-amino acid peptide is more potent antigenically compared to smaller peptides [44]. We report in this paper the results for the window size 14, and only mention the fact that our analyses with the other window sizes do not show any major variation from the results of the 14-aa long peptide stretches.

### ASA (Average Solvent Accessibility) Profiling

To extract the solvent accessibility profile we have chosen single VP7 representatives from each serotype and input them to the solvent accessibility prediction server (the SABLE server) [45]. The prediction algorithm of the server is neural network based and is reported to have a classification accuracy between 77% and 78.4% for all three states of a protein structure [45]. The server generates a single solvent accessibility index at each amino acid position. The index number ranges from 0 to 100 signifying how much of the residue is exposed to the solution with 100 being the maximum exposure number. The results (i.e., generated numbers) for each amino acid position are then averaged over window length segments to create a single average solvent accessibility at each position and a profile generated for the entire protein sequence. This is done for several sequences from the protein sequence database and an average profile obtained for the data set.

The ASA profile was further tested by submitting the same set of sequences to three other ASA prediction servers: the I-TASSER (http://zhanglab.ccmb.med.umich.edu/I-TASSER/), HHpred (PredictProtein; http://www.predictprotein.org/) and Jpred (http://www.compbio.dundee.ac.uk/www-jpred/). All the servers’ predictions generate essentially similar results, and are comparable to the SABLE results reported above; indeed, the correlation of Hhpred, jpred and I-Tasser with SABLE are 82%, 83%, 76%, respectively. The conclusions we draw from a close examination of the SABLE and the 20D results of specific regions of the lowest sequence variability and highest surface exposure thus are additionally supported.

### Combining GSWM and ASA Results

Next, as mentioned in the previous subsections, each sequence is scanned by a graphical sliding window and the results for all sequences are compared within each window to determine how many different pR values have been generated to estimate the extent of sequence variability; the numbers we get over the length of the sequence will lead to a variability profile. The solvent accessibility profile is then compared with the sequence variability profile and segments with high solvent accessibility and low sequence variability are identified as can be seen in Figure 1 (discussed later). Where 3D structures are available, e.g., 3GZT.pdb [46] and 3IYU.pdb for VP7, these identified regions are mapped onto the 3D structure of 3GZT [46] to verify that a significant portion of the selected regions are indeed surface exposed. Segments that pass these tests are then finally identified as *highly conserved surface exposed peptide segments* that could be considered for possible vaccine and/or drug development targets. These regions are then analyzed further for possible epitope like properties towards T-cell and B-cell activation through selected epitope prediction servers, discussed in more detail below. Four such regions have been identified in the VP7 protein sequence by our method and all the four peptides have shown good results as epitopic candidates.

### Epitope Prediction

While the antibody generating capability of the peptide segments identified by our technique has to be finally tested in wet labs, we have probed these stretches for presence of epitopes by using two epitope-prediction tools, viz., the IEDB (Immune Epitope Database and Analysis Resource) [47] and ABCpred servers [48], which have given good results in several applications. Cantor et al. used IEDB server for investigating immunogenic character of therapeutic enzymes [49], Singh et al. used the same IEDB server for Helper T-cell epitope mapping job in a pneumococcal surface protein [50], Gu et al used ABCpred server for characterizing phage displayed specific epitope in BALB/c mice [51], Altindis et al used the same ABCpred server for identifying epitopes in immunogenic peptides of *Bordetella Pertusis* [52], etc. We have used both of these servers to predict possible epitopic regions within the identified peptides: the IEDB server is used for predicting T-cell epitope and MHC (Major Histocompatibility Complex) binding predictions and ABCpred server for B-cell epitope prediction. Evidences for cytotoxic T-lymphocyte targets are also found on VP7 proteins [53], [54]; which makes it possible that they can also be found in the conserved surface exposed regions found through our prediction method. It is to be noted that while the IEDB server lists a large number of epitopes of the VP7, Aoki et al [19] has explicitly shown the antibody binding to and escape mutants of some of these epitopes. These are shown schematically in our Figure 2.

## Results and Discussion

The protein sequence variability and ASA profiles generated from the rotavirus sequences as described above are shown in Figure 1, the profile data plotted against amino acid positions on the sequences. The ASA profile has been scaled to the protein profile total for easy reference. The higher the points in the ASA profile, the higher is the solvent accessibility of the peptide stretch represented by the corresponding position on the sequence. Similarly, the higher is the point on the *p_R_* profile graph at any position, the more is the variability in amino acids in the sequence at that position.

Previous studies on rotavirus have already shown many regions with high variability in amino acid content and arrangement [55]. Nine variable regions in VP7 protein sequence are well known [55]. Figure 2 compares these nine variable regions (colored green) with previously recorded antigenic sites (colored red) identified by the IEDB epitope prediction server aligned by amino acid positions. In a recent study, Aoki et al [19] showed the positions of neutralizing escape mutations on rotavirus surface and about all of the data are covered by the red marked regions shown schematically in our Figure 2 (see also File S2). The schematic shows that the regions of variability partly overlap the regions of antigenic sites. Among them, variable regions VR5 (aa 87–101), VR7 (142–152) and VR8 (aa208–221) have a larger overlap and they are the major neutralizing epitopes; these have also been termed antigenic regions A, B and C respectively [56]. It is also seen from Figure 1 of the variability profiles, that all nine variable regions have significantly higher range in both variability and solvent accessibility. It is to be noted that while the protein variability profile numbers relate to *p_R_* values generated for 14 amino acid stretches at each point, the VR regions are indicated as the absolute amino acid position numbers and therefore exact matches with protein variability profile may appear slightly shifted. Because of their situations in solvent accessible and variable regions, escape mutants with single amino acid changes occur easily in case of viruses [57].

From Figure 1, comparing the relative variance between the protein variability and ASA profiles, we have identified four peptide regions as being comparatively most conserved across all serotypes and having relatively high surface accessibility. These are designated in the Figure 1 as peptide-a (aa 194 to 207), b (242 to 255), c (273 to 286) and d (301 to 314) and their structural roles are described below. In Figure 2 these four regions are indicated by blue color and two of them (peptide b and c) are found to have a considerable overlap with the previously recorded antigenic regions (red). All the four peptide regions are found to remain constant even with changes in window size in our GSWM scanning, implying that these regions contain localized conserved characteristics. Comparing the two profiles we also note that in some regions the protein profile shows very low variability and the ASA profile is also low implying that these regions are well conserved but are solvent inaccessible. These regions are actually core regions of the protein and can be so observed from the 3D structure (Figure 3). As the structural and functional roles of these peptide fragments are very important to measure their potency as antiviral candidates, we have considered also the role of the identified sequences through various means like: i) 3D structural study, ii) Epitope prediction and iii) Study of structural uniqueness.

### i) 3D Structural Study

#### Peptide-a (aa194 to aa207)

This is the first peptide from N-terminal region amongst the four peptides identified. The difference between the two profile graphs is about 20 units on the graph in the comparison scale with above mentioned particular conserved and surface exposed characteristics. In the crystallographic structure (Colored bright pink; Figure 4) we have found this region to be situated on the extracellular surface of the protein. Also, this peptide is found to be very close to the interaction point of other VP7 trimers which may explain its reason for having conserved characteristics.

#### Peptide-b (aa242 to aa255)

Second from N-terminal, this region covers a wide area of VP7 surface. In this case the difference between the two profile graphs is about 36 units which is the largest among all four varieties. In crystallographic structure this region (colored blue; Figure 5 and Figure 6) is found quite surface exposed. It also contains a special characteristic: a small portion of it traverses through the inner hydrophobic region of VP7 and as a result this segment is found to be discontinuous on the surface. This discontinuity characteristic divides the peptide into two surface exposed conserved patches, one situated on the environmental side of the protein and the other situated on the side of the VP6 interaction points. These kinds of discontinuities in surface exposed conserved segments can lead to discontinuous epitopes.

#### Peptide-c (aa273 to aa286)

This is the third peptide from the N-terminal. It contains a profile difference of around 20 units. Unlike peptide-a, this region is continuously surface exposed and from crystallographic structural evidence it can be seen that peptide-c region (Figure 7) is situated on the VP6 interaction site of rotavirus [58]. This opens the possibility of generating anti-viral molecules to act on this peptide to block its interaction with VP6 and thus render the virion ineffective. This region is also found to be very close to the surface patch of peptide-b and peptide-d, which indicates that peptide-b, peptide-d and peptide-c all together can be useful as a discontinuous epitope.

#### Peptide-d

The fourth peptide is located very near to C-terminal (Carboxy Terminal) and holds a profile difference of about 25 units. In crystallographic structure this stretch is not complete because of terminal loss of amino acids. It can be seen from Figure 5 and Figure 6 that the residues are situated in the interface region of two VP7 chain of trimeric complex raising the possibility of trimeric destabilization by interactions on this segment.

### ii) Epitope Prediction – MHC-II Binding Prediction

Though only wet laboratory and animal studies will determine whether the conserved surface exposed regions we have determined can lead to generation of immune response, we have utilized epitope prediction servers to analyze potentiality of these peptides. All four surface accessible conserved regions were submitted to the IEDB [47] epitope prediction server for T-cell MHC-II binding predictions. The results are given in **Table 2**. For each segment there are two entries because the IEDB server picked up two overlapping segments of 13 aa stretch from the 14 aa length submission. ARB (Average Relative Binding) percentile score and SMM (Stabilized Matrix Method) percentile score show that all four peptides hold quite good score for being immunogenic antigen; among them peptide stretches a and b appear to be relatively better candidates.

Additionally we have used ABCpred [48], an artificial neural network based B-cell epitope prediction server for predicting B-cell epitope on the whole VP7 protein. This server also yields positive result for the conserved surface exposed sequence (**Table 3**). It can be seen in **Table 3** that the first three ranks cover three of the previously mentioned conserved regions.

### iii) Study of Structural Uniqueness

To determine the uniqueness of the four regions identified by our procedure, i.e. to rule out the existence of a common motif, sequence or structure for these peptide segment sequences within other organisms which could lead to a false positive prediction, we have submitted peptides sequences to homology and similarity search servers. NCBI protein-protein BLAST [59] search has shown that for all these four sequences there are no matches beyond 5/14 with any other sequence except the rotavirus super family. Also, search for 3D structural alignment in PSIPRED [60] server did not yield any confirmed result for any of the four segments. Thus, these analyses indicate the uniqueness of these peptides, which is an important consideration for generation of unique immune response. Additionally they can be also used as probable candidates for drug targets.

### Conclusions

We have shown through a 20D graphical representation and numerical characterization method for protein sequences that the surface glycoprotein VP7 of the rotavirus virion posseses at least four regions that are predominantly solvent accessible and highly conserved which also have epitopic significance. Our analytical methods applied earlier in the case of the neurmainidase protein of the influenza A viruses, have been modified to avoid possible redundancies and we have checked solvent accessibilities prediction through multiple servers as well as take into account epitopic significance. The results thus indicate a high degree of probability that these four regions can be considered as suitable targets for development of peptide vaccines. The possibility remains however that in vivo configuration of the protein could show divergence [61] in surface exposure from the crystallized configuration we have used for final confirmation of our identified regions, but the primary results of our analysis that these regions are highly conserved are independent of this consideration. Though this study is purely based on using computer algorithms and a new mathematical model and has some advantages over the older method, it provides a platform to initiate preclinical studies in animal models to evaluate the vaccine potential of these conserved peptides which can be used for designing probable drugs and vaccines against the viral protein. Future wet lab studies are required to confirm our hypothesis.

Finally, we would like to make an appeal that there are many reasons to continue development of alternative vaccine candidates. Of these, the first is to avoid delay in introduction of new vaccines if for some reason current vaccines fail in developing countries due to either safety issues related to intussusception or low efficacy. The logic is to have lesser time between two vaccines, unlike when Rotashield was withdrawn in 1999; it took 6 years for Rotarix to be available in USA (2005) for vaccination. The second important reason is the cost of current vaccines. At present there is an initiative for financial support for supplementing the cost by GAVI (The Global Alliance for Vaccines and Immunization) ALLIANCE for introduction of vaccines in poor countries. But at some point of time this support will stop and then most of the third world countries will not be able to bear the current cost and continue with the vaccination programs. Thus development of alternative rotavirus vaccines, especially which can be developed and manufactured in developing nations effectively at low cost will drive down the prices due to competition in market and help reduce a scourge that leads to deaths of millions of children worldwide.

## Supporting Information

File S1 **GSWM analysis curve of VP6 and VP4.**
(XLS)

File S2 **Previously recorded VP7 epitopes from IEDB server.**
(XLS)
